# Expression and correlation of Twist and gelatinases in breast cancer

**DOI:** 10.3892/etm.2013.1099

**Published:** 2013-05-02

**Authors:** MIN ZHAO, HONG-GUANG HU, JIN HUANG, QIANG ZOU, JIN WANG, MING-QIANG LIU, YANG ZHAO, GUI-ZHU LI, SONG XUE, ZHENG-SHENG WU

**Affiliations:** 1 Department of Pathology, The Second People’s Hospital of Hefei, Hefei, Anhui 230011;; 2 Department of Pathology, The First People’s Hospital of Huainan, Huainan, Anhui 232007;; 3 Department of Pathology, Anhui Medical University, Hefei, Anhui 230032, P.R. China

**Keywords:** breast neoplasms, pathology, gelatinases, matrix metalloproteinase 2, matrix metalloproteinase 9, immunohistochemistry

## Abstract

Altered expression of Twist, matrix metalloproteinase (MMP)-2 and MMP-9 proteins has been identified in various types of human cancers. However, the correlation between Twist and these gelatinases in breast cancer remains unclear. In this study, immunohistochemical analysis of Twist, MMP-2 and MMP-9 expression was performed on tissue microarrays from 200 breast cancer cases. The association of Twist and gelatinase expression with clinicopathological factors and patient survival was analyzed. Altered expression of Twist, MMP-2 and MMP-9 proteins was observed in breast cancer tissue. The positive rates of Twist, MMP-2 and MMP-9 protein expression were 75.5, 97.0 and 96.0%, respectively. Increased expression of Twist was positively correlated with the status of axillary lymph node metastasis and higher tumor-node-metastasis (TNM) stage (P<0.01). Moreover, increased expression of Twist was correlated with poor overall survival (OS) and post-operative relapse-free survival (RFS), compared with those for the patients with reduced expression levels of Twist (P<0.05, P<0.01). The expression of MMP-2 and MMP-9 was positively correlated with Twist expression (P<0.001). Our results indicate that Twist may play an important role in the invasion, metastasis and prognosis of breast cancer. Additionally, our results suggest that Twist may be a regulator of gelatinases (MMP-2 and MMP-9).

## Introduction

Invasion and metastasis are dynamic, complex and multistep processes, and are the leading causes of mortality among breast cancer patients (1). Previous studies have suggested that matrix metalloproteinases (MMP-2 and MMP-9) and their inhibitors (TIMP-1 and TIMP-2) play important roles in the invasion and metastasis of breast cancer (2–4). Exploring the upstream regulator of MMPs in breast cancer and the underlying mechanism is important for understanding the invasion and metastasis of tumors. Twist, a basic helix-loop-helix (bHLH) transcription factor, was originally reported as a master regulator of embryonic morphogenesis (5). However, in previous studies, the Twist gene as an oncogene has been shown to play an essential role in diverse pathways, including tumor cell apoptosis, angiogenesis, invasion and metastasis, which are involved in carcinogenesis and cancer progression (6–8). To explore the functions of Twist in breast cancer and investigate whether the alteration of Twist has an effect on the expression of MMP-2 and MMP-9, we determined the expression of Twist, MMP-2 and MMP-9 proteins in 200 breast cancer tissue specimens by immunohistochemical (IHC) assay, and studied the correlation between Twist expression and clinicopathological characteristics in the breast cancer tissue samples. Moreover, we further investigated the correlation between Twist and gelatinase (MMP-2 and MMP-9) expression in breast cancer.

## Materials and methods

### Patients and specimens

The patient population consisted of 200 breast cancer patients at the Second People’s Hospital of Hefei, the First Affiliated Hospital of Anhui Medical University and the First People’s Hospital of Huainan between April 2001 and April 2002. The 200 patients had a median age of 50 years (range, 27–78 years). Patients who had undergone chemotherapy or radiation therapy prior to surgery were excluded, as were patients with rheumatic disease, acute infection, human immunodeficiency virus (HIV) or other types of cancer. The pathological tumor stage was defined according to the sixth edition of the tumor-node-metastasis (TNM) classification of the International Union against Cancer. Tumor differentiation was defined according to the 2003 World Health Organization (WHO) classification of tumors (9). Complete follow-up data were obtained from 126 breast cancer patients. Primary study endpoints were post-operative overall survival (OS) and post-operative relapse-free survival (RFS). OS and RFS were defined as the time from the date of surgery to the date of mortality from breast cancer or to the date of local recurrence or detection of distant metastasis, respectively. All tissue diagnoses were confirmed by permanent histology. A protocol for the use of tissue samples from patients and follow-up study was approved by the Institutional Review Boards of the Second People’s Hospital of Hefei, the First Affiliated Hospital of Anhui Medical University and the First People’s Hospital of Huainan. Every patient had signed a consent form.

### Tissue microarray construction

All the hematoxylin and eosin (H&E)-stained sections from each formalin-fixed, paraffin-embedded block were assessed to identify target areas. Three to five representative 1-mm cores were obtained from each case and embedded in a grid pattern into a recipient paraffin block using a tissue arrayer (Hengtai Instruments Inc., Liaoning, China). Consecutive 3-im sections were cut from the paraffin block and then attached to 10% polylysine pre-treated slides.

### IHC analyses

IHC analyses of Twist, MMP-2, MMP-9, estrogen receptor (ER), progesterone receptor (PR) and human epidermal growth factor receptor 2 (HER-2) protein expression were performed using a Two-Step Histostaining kit (Changdao Biotech Co., Ltd., Shanghai, China) with a polyclonal antibody against Twist (1:200; Santa Cruz Biotechnology, Inc., Santa Cruz, CA, USA) and monoclonal antibodies against human MMP-2 (1:200; Maixin, Fuzhou, China), MMP-9 (1:200; Maixin), ER (working solution; Changdao Biotech Co., Ltd.), PR (working solution; Changdao Biotech Co., Ltd.) and HER-2 (working solution; Changdao Biotech Co., Ltd.). The sections were deparaffinized in xylene and rehydrated in a graded series of ethanol solutions. For antigen retrieval, slides were heated in a microwave oven in 0.01 M sodium citrate buffer (pH 6.0) for 20 min. Then, the slides were allowed to cool in the same buffer and were subsequently immersed in 3% hydrogen peroxide in methanol for 10 min to block endogenous peroxidase activity. After rinsing with phosphate-buffered saline (PBS; 2 min, 3 times), slides were incubated with primary antibody at 4°C overnight. Then, slides were rinsed in PBS as above, incubated for 20 min with universal horseradish peroxidase-conjugated detection reagent (Changdao Biotech Co., Ltd.), rinsed in PBS as above, incubated with 3,3′-diaminobenzidine tetrahydrochloride (Changdao Biotech Co., Ltd.) and then all IHC slides were counterstained with hematoxylin staining solution. Known positive samples were used as positive controls. For negative controls, the primary antibody was replaced with 0.01 mol/l PBS.

### Scoring of stained sections

Immunostaining signals were reviewed and scored independently by two expert pathologists under double-blind conditions. The sum of the extent and intensity score was used as the final staining score for Twist, MMP-2 and MMP-9. The extent of staining, defined as the percentage of positively stained areas of tumor cells in relation to the whole tissue area, was scored on a scale of 0–3 as follows: 0, no staining; 1, less than one-third; 2, one-third to two-thirds; and 3, greater than two-thirds. The staining intensity was scored as 0, no staining; 1, weakly stained; 2, moderately stained; and 3, strongly stained. For the evaluation of Twist expression, a final staining score <6 was considered to be weak expression and ≥6 was considered to be high expression (10). For the evaluation of MMP-2 and MMP-9, a final staining score ≥3 was considered to be positive (11). For the evaluation of ER and PR expression, a percentage of stained tumor cells >10% was considered to be positive. For the evaluation of HER-2, membrane staining intensity and pattern were evaluated as follows: 0, completely negative or <10% of tumor cells had membrane positivity; +, >10% of tumor cells had incomplete faint membrane positivity; ++, >10% of tumor cells had complete moderate membrane positivity; and +++, >10% of tumor cells had complete strong circumferential membrane positivity (12).

### Statistical analysis

All statistical analyses were performed using SPSS software system for Windows (version 10.0; SPSS, Inc., Chicago, IL, USA). P<0.05 was considered to indicate a statistically significant difference. The Chi-square test was used to examine the difference in the positive expression rate between the groups. The correlation between the positive expression rate and the different clinicopathological parameters was examined using the non-parametric Spearman’s rank correlation analysis. Variables associated with OS and RFS rates were tested using Kaplan-Meier estimates and compared by log-rank test.

## Results

### Expression of Twist, MMP-2 and MMP-9 proteins in breast cancer specimens

**Figure 1.**
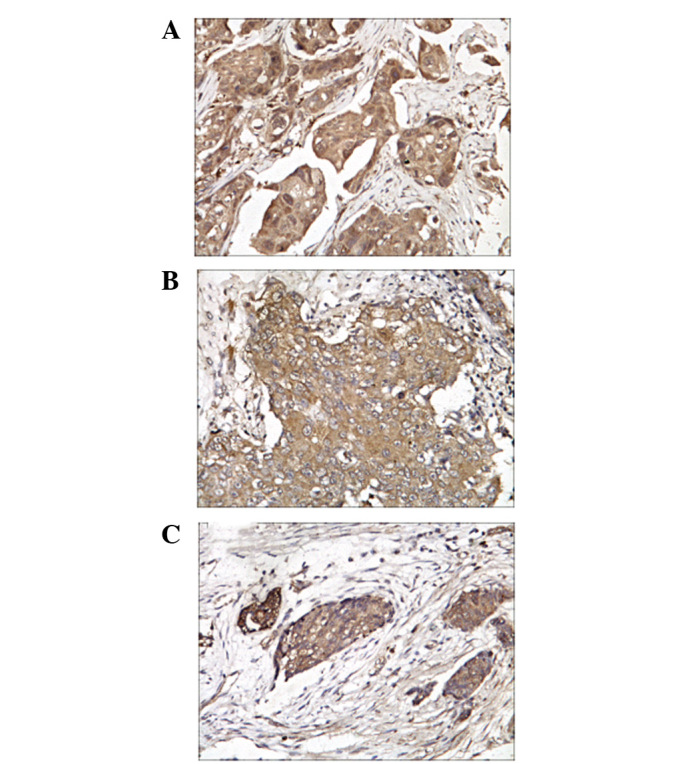
Immunohistochemical analysis of Twist, matrix metalloproteinase (MMP)-2 and MMP-9 protein expression in breast cancer. (A) Twist protein expression in the cytoplasm and nucleus of breast cancer cells; (B) MMP-2 protein expression and (C) MMP-9 protein expression in the cytoplasm of breast cancer cells. Original magnification, ×400.

The positive signals of Twist, MMP-2 and MMP-9 protein expression were predominantly located in the cytoplasm and/or nucleus of breast cancer cells (Fig. 1).

IHC analyses were performed on 200 breast cancer tissues specimens. Twist protein expression was detected in 151 (75.5%) of the breast cancer tissues, while the expression of MMP-2 and MMP-9 were detected in 194 (97.0%) and 192 (96.0%) specimens.

### Association of the expression of ER, PR, HER-2 and Twist with clinicopathological features of breast cancer

As shown in Table I, increased Twist expression was associated with increased lymph node involvement (P=0.001) and higher TNM stage (P=0.001). Twist expression was correlated with the expression of ER and PR, although these did not reach statistical significance (P=0.063 and 0.055, respectively). There was no significant association of Twist with HER-2 protein expression (P=0.745).

### Correlation between Twist expression and patient survival

**Figure 2.**
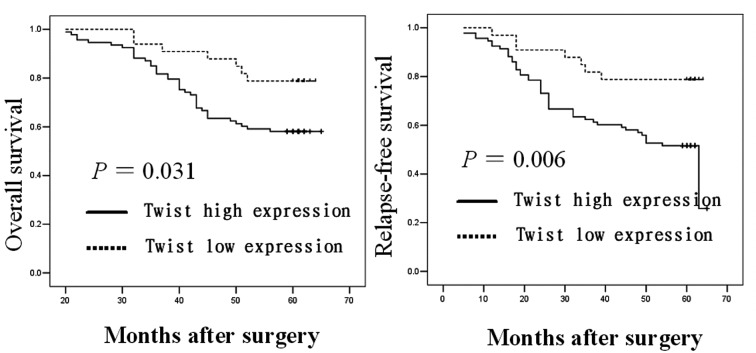
Correlation between Twist protein expression and overall survival (OS) and relapse-free survival (RFS) in breast cancer patients.

We performed Kaplan-Meier estimates and Log-rank test to determine whether the expression of Twist is associated with OS and RFS of breast cancer patients. Among the 126 breast cancer patients with complete follow-up data, those with primary tumors expressing higher levels of the Twist protein had a significantly poorer OS and RFS compared with those with lower Twist protein expression (P=0.031 and 0.006, respectively; Fig. 2).

### Association of the expression of MMP-2 and MMP-9 with clinicopathological features of breast cancer

The protein expression of MMP-2 and MMP-9 was positively associated with the status of lymph node metastasis and TNM stage (P<0.001). However, there was no significant association of MMP-2 and MMP-9 protein expression with patient age, tumor size, histological grading and ER, PR and HER-2 protein expression (P>0.05).

### Correlation of MMP-2, MMP-9 and Twist protein expression in breast cancer tissue

Spearman’s correlation analysis demonstrated that Twist protein expression was positively correlated with MMP-2 and MMP-9 protein expression (rs=0.828, P<0.001 and rs=0.500, P<0.001, respectively).

## Discussion

In the present study, we demonstrated that increased Twist, MMP-2 and MMP-9 protein expression levels are associated with increased lymph node involvement and higher TNM stage. Furthermore, Twist protein expression correlated with MMP-2 and MMP-9 protein expression in the breast cancer tissue specimens.

The gelatinases, MMP-2 (gelatinase A) and MMP-9 (gelatinase B), are two members of the MMP family and play a critical role in tumor invasion and metastasis (13,14). Several studies have demonstrated that gelatinases induce proteolytic degradation of extracellular matrix (ECM) components and basement membranes to facilitate the invasion of tumors (15–17). In the present study and a previous study (3), we demonstrated that MMP-2 and MMP-9 protein expression is associated with increased lymph node involvement and higher TNM stage. Thus, our data suggest that MMP-2 and MMP-9 may play fundamental roles in breast cancer invasion and metastasis.

Epithelial-mesenchymal transition (EMT) is a characteristic of the most aggressive metastatic cancer cells and is critical for the induction of invasiveness and metastasis of human cancers (18,19). Increasing evidence suggests that Twist acts as one of the major EMT inducers by regulating E-cadherin expression to promote cancer progression (8,20–22). Kyo *et al* (10) detected the expression of Twist in 70 cases of endometrial carcinoma and observed that 51% of the patients presented high Twist expression and the increased expression of Twist was positively associated with local tumor invasion and poor OS. Yang *et al* (23) detected Twist expression in several human breast tumor cell lines. The authors observed that invasive and metastatic cell lines expressed Twist, while non-metastatic breast tumor cell lines did not. In addition, the authors demonstrated that suppression of Twist expression inhibits tumor metastasis and reduces the presence of tumor cells in the blood circulation in a mouse model. Consistent with their results, the present study demonstrated that Twist protein expression is correlated with lymph node involvement and TNM stage, suggesting that Twist may be involved in the invasion and metastasis of breast cancer.

Moreover, our data also suggest that Twist protein expression is positively associated with gelatinase expression in breast cancer. Lee *et al* (24) identified that EMT is induced by transforming growth factor (TGF)-β and Twist in mammary epithelial cells via a MMP-dependent mechanism. Yu *et al* (25) explored the functions of Twist in hypopharyngeal cancer tissue samples by IHC assays and the results indicated that alteration of Twist has an effect on EMT, c-fos and MMP-9 expression. Luo *et al* (26) transfected the Twist gene into human gastric carcinoma MKN28 cells with a Twist sense plasmid. The authors demonstrated that the migration and invasion ability of Twist-MKN28 cells was clearly increased. Moreover, overexpression of Twist in MKN28 cells promoted the expression of cyclin D1 and MMP-2.

The current study suggests that the Twist gene may play an essential role in breast cancer invasion and metastasis. Twist may serve as a potential novel prognostic factor for breast cancer patients. Furthermore, there is a significant association of Twist and gelatinases with breast cancer progression and it is possible that Twist serves as a potential regulator of gelatinases. Further studies are required to explore the regulatory mechanisms between Twist and gelatinases.

## Figures and Tables

**Table I.** Correlation between Twist protein expression and clinicopathological parameters of breast cancer patients.

Clinical and pathological features	n	Twist, n (%)	P-value
Age (years)			0.514
≤35	21	18 (85.7)	
36–55	112	83 (74.1)	
≥56	67	50 (74.6)	
Tumor size (cm)			0.055
≤2	14	12 (85.7)	
>2–5	148	105 (70.9)	
>5	38	34 (89.5)	
Lymph node metastasis			0.001
0	69	34 (49.3)	
1–3	69	58 (84.1)	
>3	62	59 (95.2)	
Histological grading			0.483
I	18	15 (83.3)	
II	125	91 (72.8)	
III	57	45 (78.9)	
TNM stage			0.001
I–II	106	68 (64.2)	
III–IV	94	83 (88.3)	
Estrogen receptor			0.063
Negative	116	82 (70.7)	
Positive	84	69 (82.1)	
Progesterone receptor			0.055
Negative	111	78 (70.3)	
Positive	89	73 (82.0)	
HER-2			0.745
Low	135	101 (74.8)	
High	65	50 (76.9)	

TNM, tumor-node-metastasis; HER-2, human epidermal growth factor receptor 2.
